# Transcranial Alternating Current Stimulation: A Potential Modulator for Pathological Oscillations in Parkinson’s Disease?

**DOI:** 10.3389/fneur.2017.00185

**Published:** 2017-05-08

**Authors:** Wei-Peng Teo, Ashlee M. Hendy, Alicia M. Goodwill, Andrea M. Loftus

**Affiliations:** ^1^Institute for Physical Activity and Nutrition (IPAN), Deakin University, Burwood, VIC, Australia; ^2^Institute of Health and Ageing, Australian Catholic University, Melbourne, VIC, Australia; ^3^ParkC, Curtin Neuroscience Laboratory, School of Psychology and Speech Pathology, Curtin University, Perth, WA, Australia

**Keywords:** non-invasive brain stimulation, transcranial alternating current stimulation, dysfunctional neural oscillations, Parkinson’s disease, neural entrainment

## Introduction

The use of non-invasive brain stimulation (NBS) such as transcranial magnetic stimulation (TMS) and direct current stimulation (tDCS) has significantly advanced our understanding of the mechanisms underpinning motor and cognitive processes in the brain. Repetitive TMS (rTMS) and tDCS have the potential to induce bidirectional changes in cortical excitability and lasting neuroplastic effects that are dependent on the nature and parameters of the stimulation used (i.e., polarity, frequency, and intensity) (1). For instance, low-frequency rTMS induces an inhibitory effect on cortical neuronal activity over the site of stimulation, whereas high-frequency rTMS induces a facilitatory effect on cortical excitability. Similarly, anodal tDCS is capable of reducing the resting membrane threshold of cortical neurons, resulting in an increase in neuronal excitability, while cathodal tDCS produces an opposite effect.

In particular, the application of interventional forms of NBS in neurological disorders such as Parkinson’s disease (PD) has been viewed with great interest. PD is a chronic neurodegenerative condition that stems from a loss of dopamine-producing neurons in the substantia nigra within the basal ganglia (BG) (2). While the origins of PD are subcortical in nature, the BG exerts its influence on higher order cognitive and motor functions through direct and indirect cortico-subcortical projects to the cerebral cortex (2, 3). In a healthy brain, the BG acts as a prime inhibitor on a wide range of motor functions to prevent unwanted or excessive movements (2). The role of dopamine therefore acts to release inhibition, and it is this interplay between excitatory and inhibitory influence of the BG has over the motor system that produces smooth purposeful movement (4). In PD, TMS studies have showed an increased state of excitability of the primary motor cortex (M1) at rest (5) and an increased cortical demand during purposeful movements (6). A study by Ni et al. (7) further demonstrated an increased state of intracortical facilitation and reduced intracortical inhibition of the M1 that may subserve motor impairments in PD. These changes in intracortical inhibitory and facilitatory circuitry imply aberrant or maladaptive forms of neuroplasticity that may underpin motor and cognitive impairments. Indeed, dopamine is known to be a key modulator of neuroplasticity, and studies using established plasticity-inducing paradigms such as rTMS (8) and paired associative stimulation (9) have demonstrated the ability to induce neuroplastic responses only when PD patients are on medications but not off. More recently, newly developed NBS techniques such as theta-burst stimulation (TBS) have tried to simulate normal neuronal activity patterns of the hippocampus by pairing gamma frequency trains of stimuli (50 Hz) with theta oscillatory rhythms (5 Hz) (10). The rationale for using high-frequency stimulation such as TBS is perhaps related to the concept of neural entrainment, where dysfunctional cortical beta oscillation often observed in PD (see subsequent section) may be disrupted. There is evidence from deep brain stimulation recordings following high-frequency stimulation (11) and dopamine administration (12) that beta oscillatory activity is attenuated, and the emergence of gamma activity facilitates motor improvements. However, while a study by Zamir et al. (13) showed that intermittent TBS can modulate measures of cortical excitability and inhibition, Benninger et al. (14) showed no change in motor function following eight sessions of iTBS in people with PD.

## Dysfunctional Cortical Oscillations in PD

Apart from changes in intracortical excitatory and inhibitory neural circuitry, PD is associated with pathological neural oscillations that are thought to underpin motor dysfunction (15). While most of what we understand about pathological oscillations in PD comes from local field potentials recorded directly from deep brain stimulator implants, dysfunctional neural oscillations are also observed at the level of the cortex using electroencephalography or magnetoencephalography (MEG) (16). The abnormalities in temporal activity of neural oscillations include changes in the frequency distribution of neural activity as well as increases and decreases in synchronization between intra-regional (within a specific region) and inter-regional (between regions) neuronal populations (17). In particular, dysfunction in cortical beta oscillations has been implicated in motor dysfunctions associated with PD (18). In healthy individuals, cortical beta and mu oscillations are suppressed just before and during motor activity, particularly in fast-paced movements (19, 20). Other frequencies, such as gamma oscillations, have also been shown to be increased when movement is initiated, which suggests an interplay of oscillatory neural activities to support overall movement production (21). In people with moderately advanced PD, beta oscillation activity is increased just prior to movement and remains elevated during a motor task (16, 22). Additionally, studies have shown a lack of increase in gamma activity in PD, which may underpin impaired perceptual binding, coupling, and switching in movement (15, 17). Longitudinal evidence further implicates the slowing of resting-state neural oscillations, driven by an increase in slow-frequency theta and alpha waves, to cognitive declines in people with PD (23). While it is difficult to determine the role of pathological neural oscillations on specific aspects of motor and cognitive function, the current evidence strongly suggests an interplay of these pathological oscillations that overall contributes toward motor and cognitive deficits observed in PD.

## NBS in PD

Interventional forms of NBS can have a positive effect on motor and cognitive function that is likely to be driven by a change in cortical excitability of specific brain areas (24). To date, five meta-analyses of the literature have been conducted to investigate the effects of rTMS on motor function in PD (25–29), while only one systematic review has examined the effects of tDCS on motor outcomes (30). In all five meta-analyses of rTMS literature, the authors found a significant, albeit modest, improvement of motor function following rTMS. Chou et al. (27) further suggest that stimulation site and frequency as well as number of pulses are key moderators of rTMS effects on motor function. In regards to tDCS, a systematic review by Broeder et al. (30) suggests that tDCS applied to the M1 had significant effects on motor function, while tDCS over the dorsolateral prefrontal cortex elicited a modest improvement in cognitive function.

**Figure 1:**
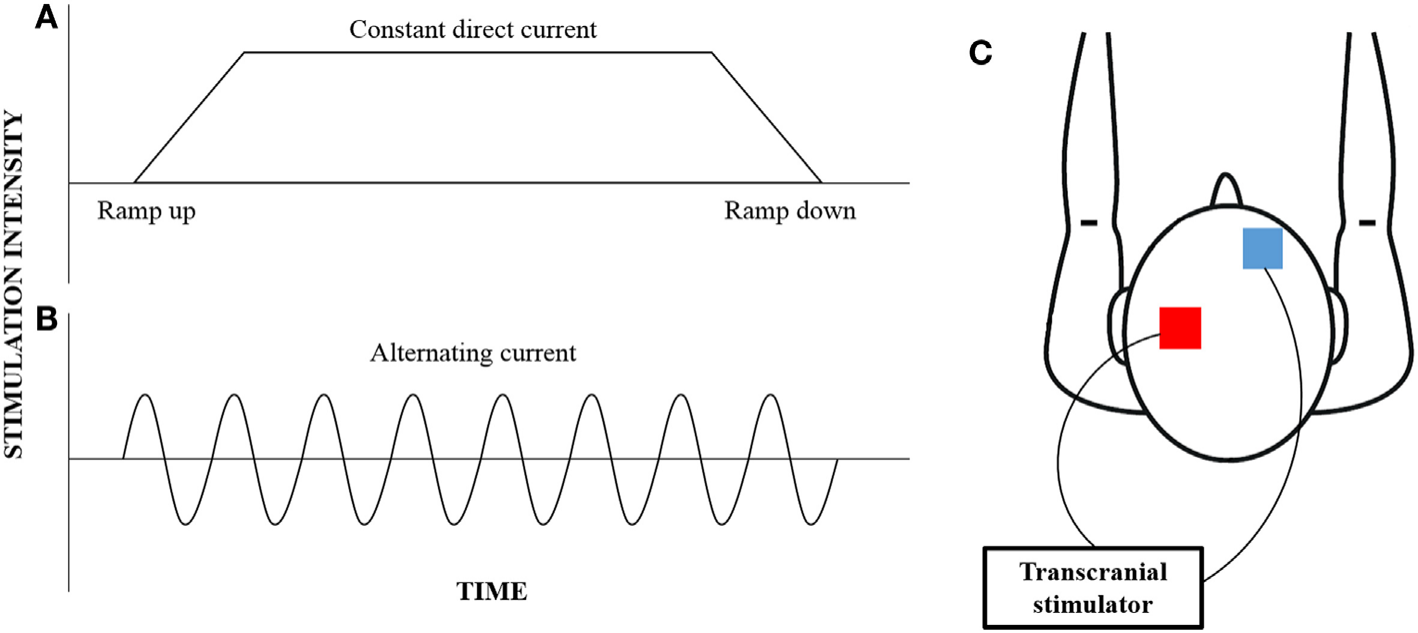
**An illustration of the different current waveforms that can be provided by transcranial stimulation**. Convention transcranial direct current stimulation **(A)** provides constant current that begins with a ramp-up phase and ends with a ramp-down phase; however, transcranial alternating current stimulation **(B)** provides a rhythmic waveform that can be customized to target specific neural oscillations. While the electrodes (red—anode; blue—cathode) are typically placed over the motor cortex and the contralateral supraorbital region **(C)**, the electrode montage can be customized to target any cortical region of interest.

Of interest, variant forms of NBS, which are frequency specific such as transcranial alternating current stimulation (tACS), are gaining increasing interest for their ability to induce lasting neuroplastic and functional change (31). tACS is a variation of the more convention tDCS, where alternating forms of electrical current at a specified frequency can be applied non-invasively through the scalp (Figure 1). While tDCS provides a constant current that can facilitate or inhibit neural excitability, tACS induces rhythmic current flow that can be used to entrain neural oscillations (32). While the concept of neural entrainment using tACS has not been thoroughly examined, pilot studies have shown that the application of tACS at a frequency corresponding to alpha oscillation frequency results in an enhancement in alpha frequency amplitude up to 30 min poststimulation (33, 34). Additionally, behavioral changes in motor and cognitive functions have also been reported following the application of tACS (35, 36). In particular, the application of tACS at beta oscillation frequency results in slowing of hand movement and reduced rate-of-force development of a hand-grip task (37), while stimulation at gamma oscillation frequency improved those parameters (38).

While there is some evidence to support the concept that neural entrainment using tACS improves motor function in healthy individuals (35, 36), the potential of tACS to improve motor function in PD remains largely untested. To the best of our knowledge, only two studies to date have investigated the effects of tACS on reducing motor symptoms in PD. The first use of tACS in PD was reported by Brittain et al. (39) aimed at reducing resting tremors in tremor-dominant PD patients. The authors used tACS to induce phase cancelation of the resting tremor rhythm. This was achieved by identifying the timing of cortical oscillations responsible for resting tremors (i.e., tremor frequency), and delivering tACS at the specific tremor frequency that would drift in and out of phase alignment with the cortical tremor frequency. The authors reported that this pioneering technique managed to achieve an almost 50% reduction in resting tremor amplitude during tACS. In a separate study by Krause et al. (40), the authors investigated the effects of 10 and 20 Hz tACS (duration—15 min; intensity—1 mA) on MEG responses during an isometric contraction of the forearm muscles and functional performance (fast finger tapping and wrist pronation–supination) in 10 people with PD and 10 healthy age-matched controls. They demonstrated that 20 Hz tACS significantly attenuated beta frequency during the isometric contraction and reduced fast finger-tapping movement amplitude variability only in people with PD.

## Conclusion

To this end, we acknowledge that the scarcity of information surrounding the effects of tACS on neural entrainment limits our interpretation of early studies in healthy individuals and people with PD. Research into the therapeutic potential of tACS is still in its early days, and there is much that is not known about the causal relationship between dysfunctions in neural oscillations and specific motor and cognitive deficits in PD. While studies by Brittain et al. (39) and Krause et al. (40) reported improved resting tremors and movement variability in PD, other cardinal motor symptoms (i.e., bradykinesia, rigidity, or gait disturbances) are likely to be driven by different underpinning mechanisms (41). However, the use of frequency-specific forms of NBS, such as tACS, may potentially represent a more targeted and individualized approach to restoring dysfunctional cortical oscillations in PD compared to more traditional forms of NBS such as rTMS or tDCS.

## Author Contributions

All the authors (W-PT, AH, AG, and AL) contributed equally to the manuscript.

## Conflict of Interest Statement

The authors declare that the research was conducted in the absence of any commercial or financial relationships that could be construed as a potential conflict of interest.
